# Visualizing the formation of an RNA folding intermediate through a fast highly modular secondary structure switch

**DOI:** 10.1038/ncomms11768

**Published:** 2016-06-13

**Authors:** Yi Xue, Brant Gracia, Daniel Herschlag, Rick Russell, Hashim M. Al-Hashimi

**Affiliations:** 1 Department of Biochemistry, Duke Center for RNA Biology, Duke University Medical Center, Durham, North Carolina 27710, USA; 2 Department of Molecular Biosciences, Institute for Cellular and Molecular Biology, University of Texas at Austin, Austin, Texas 78712, USA; 3 Department of Biochemistry, Beckman Center, Stanford University, Stanford, California 94305, USA; 4 Department of Chemistry, Stanford University, Stanford, California 94305, USA; 5 Department of Chemical Engineering, Stanford University, Stanford, California 94305, USA; 6 Chemistry, Engineering, and Medicine for Human Health (ChEM-H) Institute, Stanford University, Stanford, California 94305, USA; 7 Department of Chemistry, Duke University, Durham, Stanford, North Carolina 27710, USA

## Abstract

Intermediates play important roles in RNA folding but can be difficult to characterize when short-lived or not significantly populated. By combining ^15^N relaxation dispersion NMR with chemical probing, we visualized a fast (*k*_ex_=*k*_1_+*k*_−1_≈423 s^−1^) secondary structural switch directed towards a low-populated (∼3%) partially folded intermediate in tertiary folding of the P5abc subdomain of the ‘Tetrahymena' group I intron ribozyme. The secondary structure switch changes the base-pairing register across the P5c hairpin, creating a native-like structure, and occurs at rates of more than two orders of magnitude faster than tertiary folding. The switch occurs robustly in the absence of tertiary interactions, Mg^2+^ or even when the hairpin is excised from the three-way junction. Fast, highly modular secondary structural switches may be quite common during RNA tertiary folding where they may help smoothen the folding landscape by allowing folding to proceed efficiently via additional pathways.

## Abstract (web summary)

Short-lived RNA folding intermediates have important roles in the folding of RNA. Here, the authors combine ^15^N relaxation dispersion NMR with chemical probing to visualise one of these intermediates, and are able to show it is a secondary structural switch, that might help with folding.

## Graphical abstract

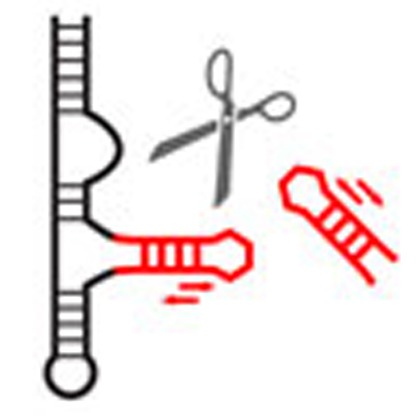

Ribonucleic acids (RNA) fold into their three-dimensional (3D) structures through a series of distinct steps and structural intermediates^1^,^2^,^3^, and detailed structural, kinetic and thermodynamic characterization of intermediates is required to elucidate RNA folding pathways. A wide variety of biophysical techniques including single-molecule fluorescence resonance energy transfer^4^, chemical probing^5^, electron microscopy^6^ and pulse-chase monitored by quantitative mass spectrometry^7^ have been developed and applied to characterize RNA folding intermediates. However, detecting intermediates and simultaneously characterizing their structure, kinetic properties and thermodynamic stabilities remain challenging, particularly when they are short-lived and/or do not populate to detectable levels.

Nuclear magnetic resonance (NMR) relaxation dispersion (RD) techniques, which quantify line broadening due to chemical exchange, have made it possible to characterize low-populated, short-lived conformational states in proteins that are often referred to as excited states (ESs)^8^,^9^. ESs have been characterized as ‘hidden' protein folding intermediates that can be difficult to detect by other methods^10^,^11^,^12^. Recently, NMR RD studies have exposed ESs in a wide variety of RNAs^13^,^14^,^15^,^16^,^17^,^18^. Transitions toward RNA ESs have been shown to often entail localized changes in secondary structure and the reshuffling of base pairs in and around non-canonical motifs such as bulges and internal loops^14^,^15^,^16^,^19^. These secondary structural transitions take place at rates that are orders of magnitude faster than larger-scale secondary structural rearrangements that typically feature more extensive remodelling of base pairs and even entire hairpins^20^,^21^. Although secondary structural transitions are common steps during the biological functions of non-coding RNA and during the assembly of ribonucleoprotein complexes, the role of ESs in RNA folding has not yet been examined^22^.

Carbon-based NMR RD techniques for characterizing RNA ESs can be difficult to apply to large tertiary RNAs, because of severe spectral overlap and greater difficulty in interpreting carbon chemical shifts, given the increase in structural complexity^16^. To address this problem, we used ^15^N *R*_1*ρ*_ RD, taking advantage of the dispersion of imino signals and better-formulated relationship between imino ^15^N chemical shifts and base-pairing type^16^,^23^. By further combining NMR RD with chemical probing and mutagenesis, we characterized ESs in the P5abc subdomain of the ‘Tetrahymena' group I intron ribozyme, an RNA for which tertiary folding pathways have been intensively studied^24^.

P5abc folds independently to form a compact tertiary structure^24^,^25^,^26^, and its folding is fast and likely precedes the folding of the P4–P6 domain of the Tetrahymena group I ribozyme and overall ribozyme folding^5^,^24^,^27^,^28^,^29^,^30^,^31^,^32^,^33^,^34^,^35^,^36^,^37^. Prior NMR^26^,^38^,^39^ and chemical probing^39^,^40^ studies showed that in the absence of Mg^2+^, a truncated P5abc construct (tP5abc) adopts an alternative secondary structure (tP5abc^Alt^), which relative to the folded state, features a single-residue register shift in base pairing within P5c, the disruption of tandem G·A pairs in P5b, and a local secondary structure rearrangement in the bulge of P5a. This alternative secondary structure does not support native tertiary folding as it sequesters residues in P5a and P5c that form tertiary contacts in the native structure, prevents formation of tandem G·A mispairs in P5b, and remodels the three-way junction topology by shortening the linker between P5c and P5a. However, tP5abc^Alt^ readily folds into a native conformation (tP5abc^Nat^) on addition of millimolar Mg^2+^ (refs 26, 38). Here, we have characterized a short-lived low-abundance tP5abc^Nat^ folding intermediate in which only P5c adopts a native-like secondary structure. This secondary structure switch occurs robustly in the absence of tertiary interactions, Mg^2+^ or even when the P5c hairpin is excised from the three-way junction.

## Results

### Mg^2+^-induced tP5abc folding monitored by NMR and SHAPE

**Figure 1:**
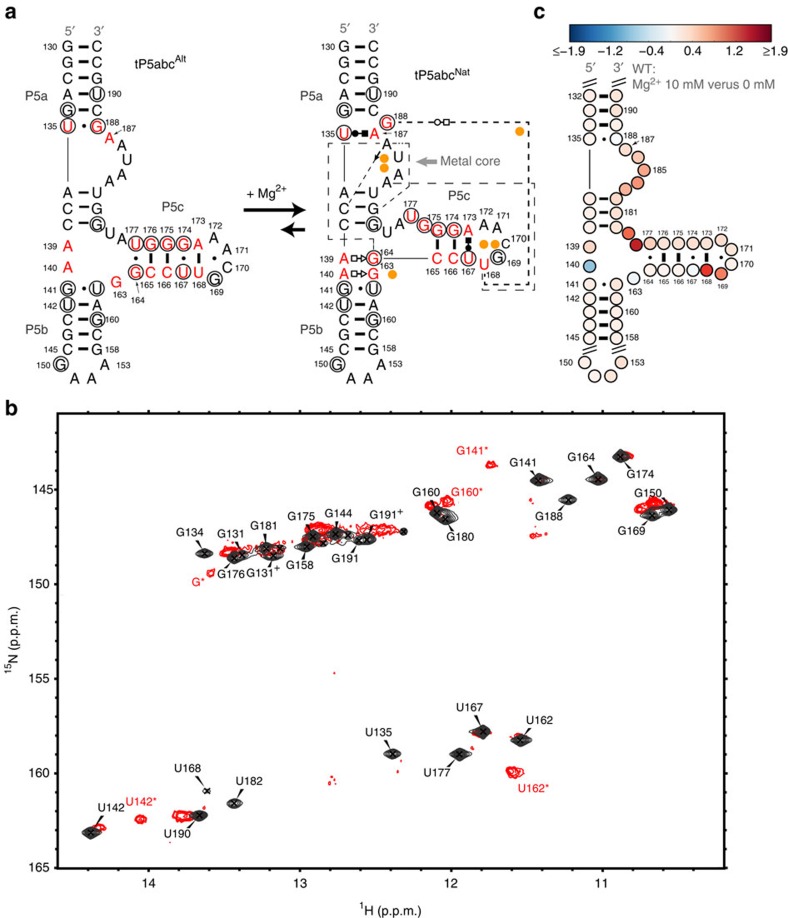
NMR and SHAPE analysis of tP5abc. (**a**) Structure of tP5abc with and without Mg^2+^. Magnesium ions identified in the X-ray structure (PDB ID: 1GID) are represented as orange spheres. Non-Watson–Crick base pairs in tP5abc^Nat^ are represented by Leontis/Westhof symbols^66^. Five base pairs are removed from P5b relative to the natural sequence. Tertiary interactions are indicated using dashed lines: U168 stacks on A183; A186 forms H-bonds with G181, C137 and G164. Residues undergoing change in secondary structure are shown in red. Residues with imino resonances that undergo changes or disappear on addition of Mg^2+^ are circled in black. (**b**) Overlay of 2D ^1^H-^15^N HSQC spectra of 1 mM tP5abc in the absence of Mg^2+^ (black) and 0.1 mM tP5abc in the presence of 7.5 mM Mg^2+^ (red) in 10 mM sodium phosphate and 0.01 mM EDTA, pH 6.4, at 10 °C. MgCl_2_ was directly added to the NMR sample. Resonances belonging to tP5abc^Nat^ are labelled with asterisks based on the previous assignments^26^. The resonances labelled with ‘+' represent alternative states for residues G131 and G191 due to minor N+1 transcripts. (**c**) Differential SHAPE reactivity of 0.2 μM WT P5abc in the presence of 10 mM Mg^2+^ relative to no Mg^2+^ conducted in 10 mM sodium phosphate and 0.01 mM EDTA, pH 6.4, at 10 °C. Red (blue) represents more (less) reactivity of one state relative to the other. The extended P5abc construct is shown in Supplementary Fig. 2a.

We used NMR spectroscopy to characterize ESs in tP5abc (Fig. 1a) that might represent rapidly forming intermediates during tertiary folding. 2D NOESY experiments on uniformly ^13^C-/^15^N-labelled tP5abc in the absence of Mg^2+^ (Supplementary Fig. 1a) allowed assignment of all imino resonances (Fig. 1b), and the results were in excellent agreement with previously reported assignments and the secondary structure of tP5abc^Alt 26^. The addition of Mg^2+^ resulted in the disappearance of imino resonances unique to tP5abc^Alt^, and appearance of a second set of resonances in slow exchange on the NMR timescale that can be assigned to tP5abc^Nat^ (ref. 26; Fig. 1b). For example, we observed the disappearance of imino resonances belonging to G188·U135 in P5a and G164·U177, G174·U167 and G169 in P5c, and the appearance of new resonances most of which have been assigned to specific residues in P5b (ref. 26; shown by asterisks, Fig. 1b). While higher Mg^2+^ concentrations are required for tertiary folding, the spectra exhibited severe line broadening under these concentrations as reported previously^26^ most likely due to non-specific aggregation at the high RNA concentrations (>10 μM) used for NMR studies. Aggregation was also observed with a full-length P5abc construct.

We therefore used selective 2′-hydroxyl acylation analysed by primer extension (SHAPE) footprinting experiments^41^ to independently monitor the conformational transition at higher Mg^2+^ concentrations (10 mM) in full-length P5abc (Fig. 1c and Supplementary Fig. 2). We observed the expected changes in reactivity on addition of Mg^2+^, including enhanced reactivity at U168 and G169, consistent with a change in P5c secondary structure, and protection at A140, consistent with formation of the tandem G·A pairs in P5b (Fig. 1c). Taken altogether, the NMR and SHAPE data strongly support the previously proposed Mg^2+^-induced folding transition in P5abc.

### Chemical exchange in tP5abc

**Figure 2:**
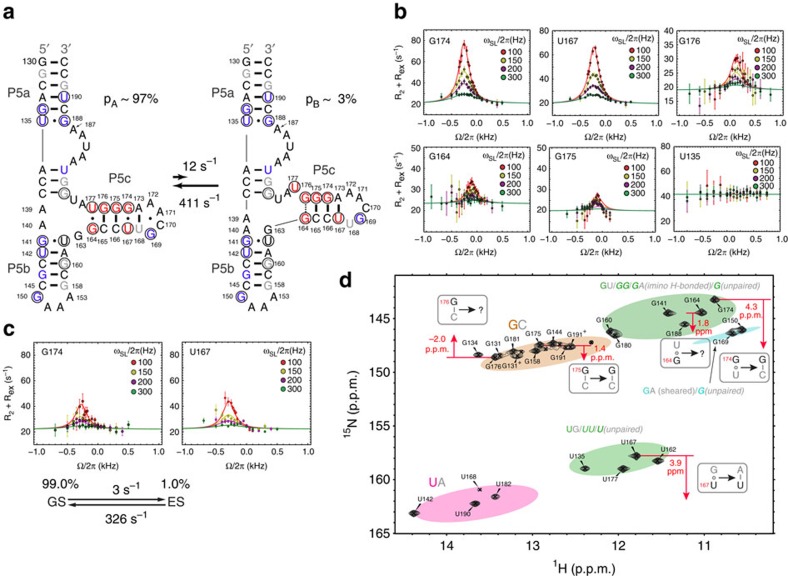
RD analysis of excited state in tP5abc. (**a**) Chemical exchange with proposed excited state that features partially folded P5c. Exchange parameters are obtained from global fitting the RD profiles. Residues are coloured as follows: RD detected, red; no RD detected, blue; immeasurable due to severe overlap or weak intensity, grey; and all others, black. Residues with imino resonances that undergo changes or disappear on addition of Mg^2+^ are circled. (**b**,**c**) Off-resonance RD profiles in the absence (**b**) and presence (**c**) of 1 mM Mg^2+^. The complete data set is shown in Supplementary Fig. 3. The data in **b** and **c** are globally fitted using two-state exchange model and Laguerre equation^61^ (see Supplementary Tables 2 and 3). (**d**) 2D ^1^H-^15^N HSQC spectrum of 1 mM WT tP5abc in 10 mM sodium phosphate and 0.01 mM EDTA, pH 6.4, at 10 °C. The residues showing pronounced RD are highlighted by red arrows starting at ^15^N chemical shift of GS and ending at ^15^N chemical shift of ES, and are labelled with the associated Δω. Also shown is the ellipsoid distribution of ^15^N (N1/3) and ^1^H (H1/3) chemical shifts of RNA imino groups in different base pair contexts. The chemical shift data are extracted from the BMRB database. The ellipses are centred on the average chemical shift and have size that corresponds to twice the s.d. in the distribution.

We used the 1D low-spin-lock field ^15^N experiment^42^,^43^,^44^ to measure RD in imino nitrogen of guanine (N1) and uridine (N3) residues in tP5abc in the absence of Mg^2+^. Significant RD indicative of slow millisecond timescale conformational exchange with a transient low-populated ES (Fig. 2a,b and Supplementary Fig. 3a) was observed for residues within the P5c stem (Fig. 2a). These residues also undergo changes in secondary structure (Fig. 1a) and ^15^N chemical shifts (Fig. 2a) on Mg^2+^-induced tertiary folding. In contrast, flat RD profiles were observed for all other residues in P5a and P5b, including residues that undergo large changes in secondary structure and ^15^N chemical shifts on Mg^2+^-induced tertiary folding (Fig. 2a,b and Supplementary Fig. 3). A two-state^9^ fit of the RD profiles measured in P5c (Fig. 2b and Supplementary Fig. 3) allowed determination of the ES population (p_B_), the forward (*k*_1_) and backward (*k*_−1_) rate constants, and the difference in chemical shift between the GS and the ES (Δ*ω*=*ω*_ES_−*ω*_GS_). Very similar exchange parameters were obtained for various residues in P5c (*p*_B_∼1.4−3.2% and *k*_ex_=*k*_1_+*k*_−1_=386−528 s^−1^). This concordance suggests a concerted exchange process directed to a single ES (global fit yields *p*_B_=2.9% and *k*_ex_=423 s^−1^) that affects all P5c residues simultaneously. Such a concerted exchange processes is consistent with a secondary structural rearrangement that simultaneously affects several residues^14^,^15^,^16^,^19^.

Despite the Mg^2+^-induced aggregation, we were able to measure RD for G174 and U167 in tP5abc in the presence of 1 mM free Mg^2+^ (Supplementary Fig. 4). Under these conditions, two sets of resonances are observed corresponding to a major unfolded tP5abc GS (∼92%) and minor fully folded tP5abc^Nat^ (∼8%) that exchange slowly on the NMR timescale (*k*_ex_=*k*_1_+*k*_−1_<1 s^−1^) (ref. 38). For both G174 and U167, we observed significant RD in the presence of Mg^2+^ (Fig. 2c). Two-state analysis of the RD profile yielded ES chemical shifts very similar to those observed in the absence of Mg^2+^ (Δ*ω*_G174_=3.9±0.4 p.p.m. and Δ*ω*_U167_=4.1±0.3 p.p.m. compared with 4.3±0.1 p.p.m. and 3.9±0.1 p.p.m. in the absence of Mg^2+^), a comparable exchange rate (*k*_ex_=*k*_1_+*k*_−1_≈329±110 s^−1^ versus 423±26 s^−1^ in the absence of Mg^2+^) and a lower ES population (1.0±0.3% versus 2.9±0.2% in the absence of Mg^2+^; Supplementary Tables 2 and 3). Standard errors were estimated by Monte Carlo simulation during fitting of RD data (see the ‘Methods' section). The lower ES population and, possibly, a slightly slower exchange result in a smaller degree of RD in the presence versus absence of Mg^2+^ (Fig. 2b,c). These data indicate that Mg^2+^ preferentially stabilizes the tP5abc^Alt^ GS relative to the ES.

The above results indicate that in the presence of 1 mM Mg^2+^, the tP5abc^Alt^ GS slowly exchanges (*k*_ex_<1 s^−1^) (refs 38, 40) with ∼8% folded state (tP5abc^Alt^⇌tP5abc^Nat^) and also undergoes a second exchange process (tP5abc^Alt^⇌ES) that is at least two orders of magnitude faster (*k*_ex_=329 s^−1^) and is directed toward a lower abundant ES (1%). This ES features a distinct structure for P5c relative to that in tP5abc^Alt^.

### An ES with partially folded P5c

One possibility is that the ES represents a partially folded intermediate in which only P5c (but not P5a or P5b) adopts a native-like secondary structure—that is, P5c undergoes the single-nucleotide register shift to adopt a native-like secondary structure without switching P5a or P5b (Fig. 2a). This would explain the localization of RD specifically to P5c residues but not to P5a or P5b (Fig. 2a, see nucleotides in red). To test this hypothesis, we analysed the ES ^15^N chemical shifts (*ω*_ES_), which are exquisitely sensitive to hydrogen bonding type, and in ideal cases, can be used to infer the nature of the base pairing^16^,^23^. The proposed switch in P5c secondary structure is expected to change base pairs and give rise to specific changes in ^15^N chemical shifts that can be directly tested by examining the ES chemical shifts.

Indeed, we found that the ES chemical shifts are consistent with a native-like P5c secondary structure (Fig. 2d). For example, the large ^15^N chemical shift change observed for G174, Δ*ω*_G174_=*ω*_ES_−*ω*_GS_=+4.3 p.p.m., is consistent with replacement of a G174·U167 mispair in the GS with a canonical WC G174–C166 base pair in the ES. Similarly, Δ*ω*_U167_=+3.9 p.p.m. is consistent with formation of a WC A173–U167 base pair, although in the X-ray structure of P4–P6 (ref. 24) this base pair is distorted, possibly due to the formation of Mg^2+^-mediated tertiary contacts. Δ*ω*_G176_=−2.0 p.p.m. is consistent with a transition from a WC G176–C165 base pair in the GS to an unpaired G176 in the ES. Δ*ω*_G175_=+1.4 p.p.m. is consistent with retention of a G–C WC base pair and a G175–C166→G175–C165 transition. While Δ*ω*_G164_=+1.8 p.p.m. is consistent with G164·U177→G164·A139, the absence of RD in residues immediately below the tandem G·A pairs (for example, U162 which shows large change in ^15^N chemical shift on transition to tP5abc^Nat^; Fig. 2a) indicates that the tandem G·A pairs in P5b, which includes G163–A140, are not formed in the ES. Rather as we discuss below, Δ*ω*_G164_=+1.8 p.p.m. likely reflects formation of a non-native G164·G176 base pair that caps the P5c helix in the ES (Fig. 2a). While we observed marginal RD at U177 as expected for a transition U177·G164→U177(unpaired), the small Δ*ω*_U177_ could not be determined reliably (Supplementary Fig. 3a). Finally, we note that while one would expect RD for U168, which transitions between a U–A WC base pair in the GS and a bulged-out U in the native state, its resonance was too weak to allow reliable RD measurement.

### NMR and SHAPE analysis of GS-stabilizing mutant

We used MCSF (mutate and chemical shift fingerprint) strategy^14^,^16^ to further test the proposed ES structure (Fig. 2a). In this approach, a mutation is used to stabilize the ES and/or GS, and the consequences of this perturbation on NMR chemical shifts, ES population and Mg^2+^-induced folding is evaluated.

**Figure 3:**
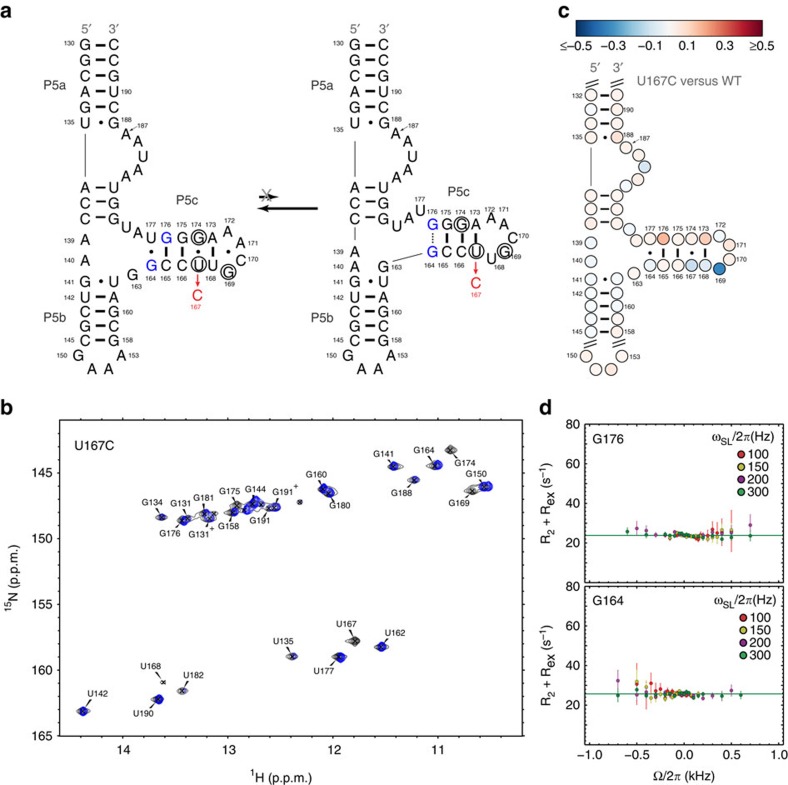
Analysis of the GS-mutant tP5abc^U167C^. (**a**) The GS/ES transition is abolished in the GS-stabilizing mutant tP5abc^U167C^. Residues disappearing or showing large differences in chemical shifts relative to WT are circled in black. Residues G164 and G176 are coloured in blue, indicating no appreciable RD. (**b**) Corresponding 2D ^1^H-^15^N HSQC spectrum of tP5abc^U167C^ in 10 mM sodium phosphate and 0.01 mM EDTA (pH 6.4) at 10 °C (blue) overlaid on spectrum of WT tP5abc under the same condition (black). (**c**) Differential SHAPE reactivity measured on tP5abc^U167C^ relative to WT. Red (blue) represents more (less) reactivity, respectively. The data were collected in the absence of Mg^2+^. (**d**) Off-resonance *R*_1*ρ*_ RD profiles measured in tP5abc^U167C^ in the absence of Mg^2+^.

We stabilized the tP5abc^Alt^ GS using a previously described U167C mutant^39^. This mutation replaces a GS G174·U167 mispair with a more stable canonical WC G174–C167 base pair, and conversely, replaces an ES WC A173–U167 base pair with a less-stable A173·C167 mispair (Fig. 3a). 2D NOESY connectivities (Supplementary Fig. 1b) and overlaid 2D ^1^H-^15^N HSQC spectra (Fig. 3b) along with SHAPE analysis (Fig. 3c and Supplementary Fig. 2b) provided evidence that the tP5abc^U167C^ mutant adopts the tP5abc^Alt^ GS secondary structure, as reported previously^39^. The 2D ^1^H-^15^N HSQC spectra of tP5abc^U167C^ were very similar to those of wild-type (WT) tP5abc (Fig. 3b). We observed the expected loss of imino resonances belonging to G174·U167 and the appearance of a new resonance that overlaps with G144 and can be assigned to G174–C167 (Supplementary Fig. 6a). Interestingly, we also observed an upfield shifted imino ^1^H resonance of G169 that likely corresponds to perturbations in the sheared G169–A172 mispair (Fig. 3b and Supplementary Fig. 6a), possibly reflecting local changes in the GCAA tetraloop due to stabilization of the stem.

If our proposed ES structure is correct, the tP5abc^U167C^ mutation would stabilize the GS relative to the ES and thereby reduce the ES population and diminish RD. Indeed, the mutation abolished all of the previously observed RD in tP5abc, including that observed at residues three base pairs away from the site of mutation (Fig. 3d). This observation provides further evidence that residues in P5c undergo a concerted exchange process directed to a single ES.

### NMR and SHAPE analysis of ES-stabilizing mutant

**Figure 4:**
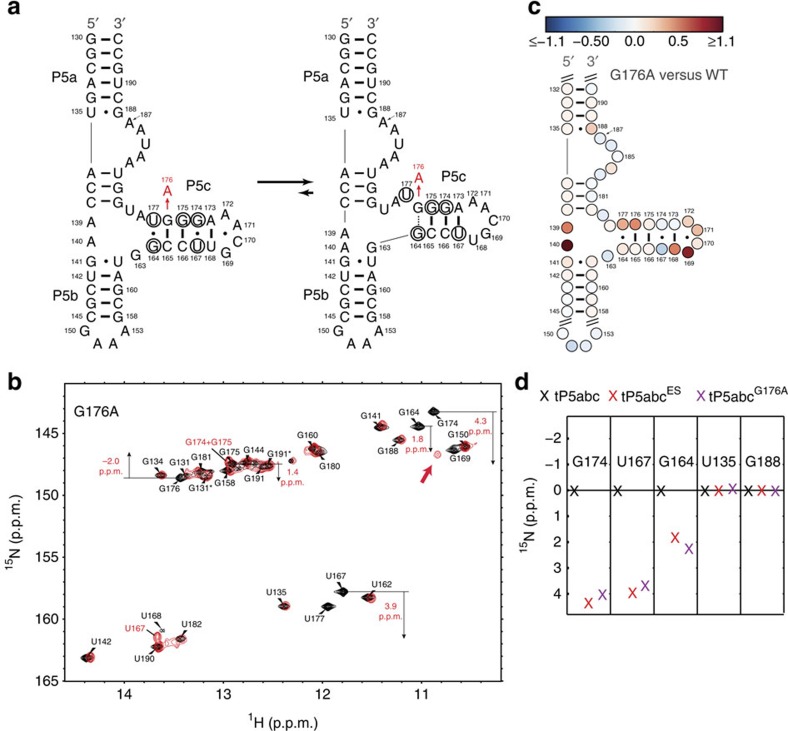
Analysis of the ES-mutant tP5abc^G176A^. (**a**) Stabilizing the ES using the tP5abc^G176A^ mutant. Residues disappearing or showing large differences in chemical shifts relative to WT are circled in black. (**b**) 2D ^1^H-^15^N HSQC spectrum of 2 mM tP5abc^G176A^ in 10 mM sodium phosphate and 0.01 mM EDTA (pH 6.4) at 10 °C (red) overlaid on spectrum of WT tP5abc under the same condition (black). The residues showing pronounced RD in WT tP5abc are highlighted by black arrows starting at ^15^N chemical shift of GS and ending at ^15^N chemical shift of ES, and are labelled with the associated Δω. An unassigned guanine resonance in the sheared G·A region is highlighted by thick red arrow. (**c**) Differential SHAPE reactivity measured on tP5abc^G176A^ relative to WT. Red (blue) represents more (less) reactivity, respectively. The data were collected in the absence of Mg^2+^. (**d**) Comparison of ^15^N chemical shifts for several residues in WT, ES and tP5abc^G176A^.

Conversely, we used the G176A mutation to stabilize the ES conformation (Fig. 4a). This mutation replaces a canonical G176–C165 WC base pair in the GS conformation with a weaker non-canonical A176·C165 mispair while minimally affecting the ES stability. If the proposed ES conformation is correct, such a mutation will likely trap an ES secondary structure with native P5c, while leaving P5a and P5b in their alternative conformations, and yield GS ^15^N chemical shifts that are consistent with those measured for the ES using RD in the parent construct. Indeed, the 2D NOESY connectivities (Supplementary Fig. 1c), 2D ^1^H-^15^N HSQC spectra (Fig. 4b) and SHAPE data (Fig. 4c and Supplementary Fig. 2b) show that in tP5abc^G176A^, P5a and P5b adopt conformations similar to those in tP5abc^Alt^, while P5c adopts a native-like conformation, consistent with the proposed ES conformation (Fig. 4a). This provides additional supporting evidence that P5c can switch independently of P5a and P5b.

Comparison of 2D ^1^H-^15^N HSQC imino spectra of uniformly ^13^C-/^15^N-labelled tP5abc^G176A^ with tP5abc^Alt^ revealed large changes in chemical shifts only within P5c, consistent with a conformational switch that is specifically localized to P5c (Fig. 4b). In the spectrum, we observed all of the expected P5c spectral changes, including disappearance of the imino resonances for U168, G169 and U177 that are unpaired in ES and large changes in the ^15^N chemical shifts for residues G174 and U167 that shift in direction and magnitude toward the chemical shifts assigned to the ES using RD measurements in tP5abc. G175 could not be unambiguously assigned and either falls in the WC region where it overlaps with other resonances or is unobservable due to solvent exchange. The imino ^15^N chemical shift of U167 is shifted toward the WC A–U region, consistent with the ES chemical shifts (Fig. 4b). The resulting P5c ^15^N chemical shifts in tP5abc^G176A^ are in excellent agreement with the ES chemical shifts measured in the parent construct (Fig. 4d).

We also observed a guanine resonance in tP5abc^G176A^ that falls in the sheared G·A region (Fig. 4b). This resonance does not show any NOESY cross peaks and was therefore difficult to assign (Supplementary Fig. 1c). Assignment to a G·A mispair in P5b is unlikely since we do not observe RD in this region in WT tP5abc. In addition, the U142 resonance in tP5abc^G176A^ undergoes a large chemical shift perturbation on addition of Mg^2+^ (Supplementary Fig. 5c), indicating that the tandem G·A pairs in P5b (Fig. 1a) are not formed in the absence of Mg^2+^. This resonance could represent an A176·G164 mispair involving the mutated residue that caps the P5c stem (Fig. 4a). An analogous non-native G164·G176 base pair capping P5c could form in the ES and would be consistent with the ES chemical shifts measured for both G164 and G176 (Fig. 2d).

The SHAPE data also provided insights into the structure of ES-stabilizing mutant P5abc^G176A^ (Fig. 4c and Supplementary Fig. 2b) and further support for the conclusions above. Consistent with the single-nucleotide register shift in P5c (Fig. 4a), U168, G169, G176 and U177 show enhanced SHAPE reactivity relative to WT P5abc, although the reactivity in U168 and G169 is complicated by signals from reverse transcriptase stopping. Similarly, A139 and A140 become further enhanced relative to WT P5abc, arguing against formation of the tandem G·A pairs. The protection pattern elsewhere is minimally affected, including the metal core region (Fig. 1a), consistent with P5c acting as an independent switch in the ES of P5abc.

### Folding from the ES intermediate is energetically downhill

**Figure 5:**
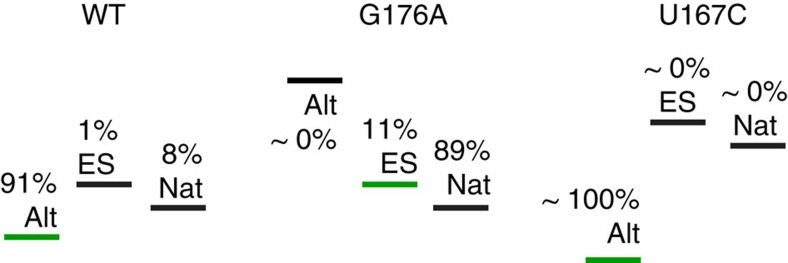
Free-energy diagrams of tP5abc (left), tP5abc^G176A^ (middle) and tP5abc^U167C^ (right) in the presence of 1 mM Mg^2+^ at 10 °C. Energy bars coloured in green indicate that they are also the GSs in the absence of Mg^2+^. All states are labelled with their associated populations.

For WT tP5abc in 1 mM Mg^2+^ at 10 °C, the population of the ES intermediate is ∼1% and is less stable than the GS by ΔG_ES-Alt_∼2.5 kcal mol^−1^. By comparison, under the same conditions, the population of folded tP5abc^Nat^ is ∼8% corresponding to ΔG_Nat-Alt_∼1.4 kcal mol^−1^. This implies that under these conditions, the transition from the ES to tP5abc^Nat^ is energetically downhill (ΔG_Nat-ES_=−1.1 kcal mol^−1^), while the transition from tP5abc^Alt^ to either ES or tP5abc^Nat^ is energetically uphill (see Fig. 5 for energy diagrams). Assuming ES can productively fold into tP5abc^Nat^, this predicts that any mutation stabilizing the ES without affecting key elements required for folding and Mg^2+^ interactions should require lower Mg^2+^ concentrations to undergo tertiary folding. In particular, under conditions of 1 mM Mg^2+^, we can predict a free-energy difference between the ES intermediate and folded tP5abc^Nat^ state of 1.1 kcal mol^−1^ which translates into *K*_fold_(G176A)≈*K*_ES,Nat_=8 for the G176A ES-stabilizing mutant.

Indeed, NMR measurements indicate that the tP5abc^G176A^ mutant requires lower Mg^2+^ concentrations to undergo tertiary folding as compared with WT (Supplementary Fig. 5). These results are consistent with prior studies showing that mutations stabilizing native P5c help stabilize tP5abc^Nat^ (refs 39, 40). More specifically, based on the peak intensities for tP5abc^G176A^ in 1 mM Mg^2+^ (Supplementary Fig. 5c), we estimate *K*_fold_(G176A)∼3, which is significantly larger than *K*_fold_(WT)=0.09 and in reasonably good agreement with the predicted value of *K*_fold_(G176A)=8. This suggests that the G176A mutation does not substantially alter the relative stabilities of ES and tP5abc^Nat^, that is, *K*_ES,Nat_(WT)≈*K*_ES,Nat_(G176A) (Fig. 5).

Conversely, we did not observe Mg^2+^-induced tertiary folding or P5c switching for the tP5abc^U167C^ mutant (Supplementary Fig. 5b) using NMR even when increasing the Mg^2+^ concentration to 50 mM, consistent with uphill folding and prior studies^39^ (Fig. 5). This result also shows that switching of P5b and P5a is less thermodynamically favourable when P5c is in the alternative conformation, consistent with prior studies^39^,^40^,^45^.

Taken altogether, these results indicate that the folding from the ES intermediate is thermodynamically downhill. Our results above also indicate that the ES is formed rapidly enough to be on the kinetic folding pathway—that is, it is formed much faster than overall folding; nevertheless, other kinetic pathways for folding are possible, as elaborated in the ‘Implications for tP5abc tertiary folding' subsection below.

### P5c exchange is modular and independent of tertiary context

**Figure 6:**
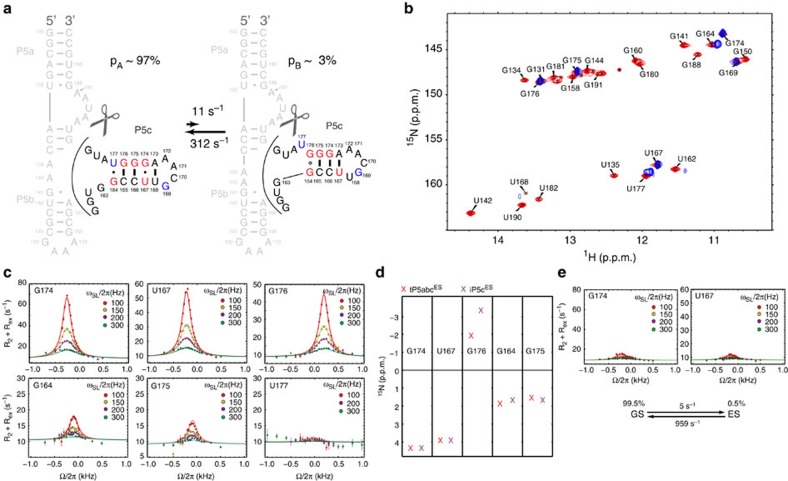
Modular exchange in P5c. (**a**) Secondary structure of isolated P5c construct (iP5c). Residues showing RD are coloured in red, residues showing no RD in blue, and those without observable imino resonances in 2D HSQC spectra in black. Exchange parameters are obtained from global fitting the RD profiles using two-state exchange model and Laguerre equation. (**b**) 2D ^1^H-^15^N HSQC spectrum of 1 mM iP5c (blue) overlaid on spectrum of WT tP5abc (red) in 10 mM sodium phosphate and 0.01 mM EDTA (pH 6.4) at 10 °C. (**c**) Off-resonance *R*_1*ρ*_ RD profiles of iP5c in the absence of Mg^2+^. (**d**) Comparison of ES chemical shifts measured in iP5c and tP5abc. (**e**) Off-resonance RD profiles of iP5c in the presence of 1 mM free Mg^2+^.

Our results show that P5c can switch efficiently to adopt a native-like secondary structure in the absence of Mg^2+^ or tertiary interactions. We examined whether an isolated P5c construct (iP5c) that is excised away from the P5abc three-way junction can retain secondary structure switching despite the absence of tertiary context (Fig. 6a). In addition to the P5c hairpin, iP5c contains five terminal residues in accordance with the tP5abc sequence (three residues at the 3′-end and two residues at the 5′-end). Two additional guanines were used to cap the 5′-end to optimize the *in vitro* transcription reaction (Fig. 6a).

We assigned NMR spectra of iP5c using 2D NOESY experiments (Supplementary Fig. 1d). We observed the expected resonances belonging to the P5c base pairs within the hairpin but did not observe clear imino resonances for the additional residues at the 3′- and 5′-end, indicating that they do not form stable base pairs (Fig. 6b). Spectra of iP5c overlay very well with those of tP5abc, and the differences observed were minor and localized at the terminal G164·U177 base pair near the site of detachment (Fig. 6b). Thus, iP5c forms a GS structure very similar to that of P5c in tP5abc.

Strikingly, we observed very similar ^15^N RD profiles in iP5c as compared with tP5abc (Fig. 6c). In particular, we observed significant RD for residues G164, U167, G174, G175 and G176, in perfect agreement with the results obtained for tP5abc (Figs 2b and 6c). A two-state fit of the RD profiles in iP5c yielded exchange parameters that are in excellent agreement with those obtained in tP5abc (*k*_ex_=323±9 s^−1^ compared with 423±26 s^−1^ in tP5abc and p_B_=3.4%±0.1% compared with 2.9%±0.2% in tP5abc). The ES chemical shifts obtained for iP5c are also in excellent agreement with those obtained for tP5abc (Fig. 6d and Supplementary Table 2). The similar exchange parameters observed for iP5c and tP5abc suggests that the non-canonical residues outside the basic helical stem do not significantly contribute to differences in energetic stability of the GS and ES.

Remarkably, the addition of 1 mM Mg^2+^ also resulted in changes in the RD and exchange parameters (Fig. 6e and Supplementary Table 3) that mirror the effects Mg^2+^ has on exchange in tP5abc (Fig. 2c and Supplementary Table 3). We observed diminished RD and an ES with similar chemical shifts (Δ*ω*_G174_=3.3±0.2 p.p.m. and Δ*ω*_U167_=2.5±0.1 p.p.m. compared with 4.3±0.1 p.p.m. and 3.9±0.1 p.p.m. in absence of Mg^2+^) and lower population (0.5±0.1% as compared with 3.4±0.1% in the absence of Mg^2+^) (Supplementary Tables 2 and 3). The higher-quality RD data in iP5c allow us to resolve a Mg^2+^-induced increase in exchange rate (*k*_ex_=964±67 s^−1^ as compared with 323±9 s^−1^ in the absence of Mg^2+^). Thus, iP5c also recapitulates the effects of Mg^2+^ on exchange observed in tP5abc, and the results suggest that the effects of Mg^2+^ on exchange in tP5abc are very likely due to interactions with P5c residues. Indeed, the addition of 1 mM Mg^2+^ to iP5c resulted in reduced line broadening of imino resonances belonging to U167/G174/G176, suggesting stabilization of P5c stem, possibly due to the interaction between Mg^2+^ ions and GCAA tetraloop. This GS-stabilization may at least partially explain the reduced population of ES in the presence of Mg^2+^. Interestingly, we did not observe iP5c switching even on addition of 30 mM Mg^2+^ (Supplementary Fig. 6b), indicating that Mg^2+^-induced P5c switching is coupled to tertiary interactions involving other structural elements in P5abc (ref. 40).

Unlike in P5abc, the G176A mutation did not lead to clear stabilization of the ES in iP5c. Rather this iP5c mutant yielded a spectrum that is consistent with multiple secondary structural species and in which imino resonances are extensively broadened. Presumably these competing secondary structures are not as favourable within the context of tP5abc and/or involve the 5′- or 3′-residues added to iP5c. Nevertheless, we were able to stabilize the ES in iP5c using a construct containing a UUCG apical loop (Supplementary Fig. 6c). This mutant allowed us to observe ES resonances (U167, G174, G175 and U177) including the potential non-native G164·G176 mispair (Supplementary Fig. 6c). The ^15^N chemical shift difference of G175-N1 between iP5c and its ES-trapped mutant is highly consistent with Δ*ω* values that were derived from RD measurements on both iP5c and tP5abc (Supplementary Table 4). This comparison could not be made using the tP5abc^G176A^ mutant, because G175 cannot be assigned unambiguously in tP5abc^G176A^ and because the point mutation at G176 introduces significant chemical shift perturbation to the G175 residue.

### Implications for tP5abc tertiary folding

The rates of P5c switching measured in the presence of 1 mM Mg^2+^ (*k*_ex_=329±110 s^−1^) are more than two orders of magnitude faster than Mg^2+^-induced tertiary folding of tP5abc (*k*_obs_=*k*_obs,f_+*k*_obs,r_=0.3–1 s^−1^) measured under similar conditions using ZZ-exchange NMR^38^ and 2-aminopurine fluorescence^40^. The observed fast P5c switching is consistent with previous studies that concluded that P5c switching is not rate-limiting to tP5abc tertiary folding^40^. The low abundance of native P5c in the absence of tertiary interactions is also consistent with prior studies indicating that stable P5c switching is coupled to the formation of tertiary interactions^40^.

**Figure 7:**
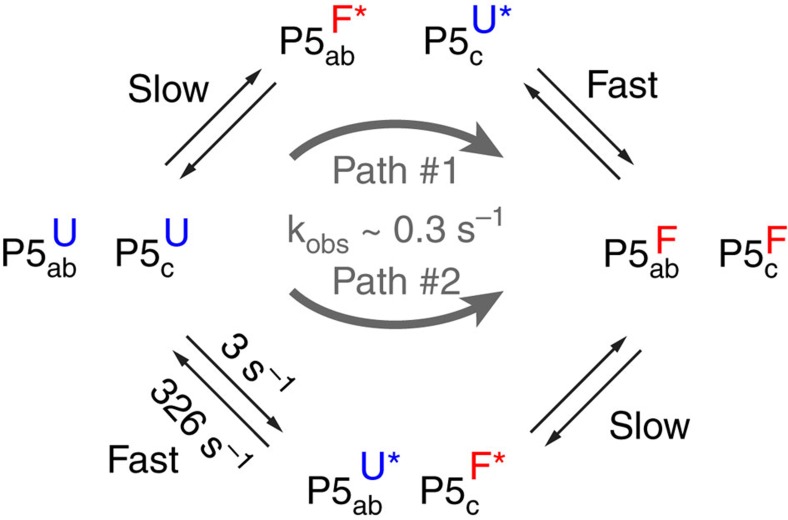
Proposed tertiary folding pathways for tP5abc involving independent P5c switching. The ‘U' and ‘F' superscript represents unfolded and folded species, respectively. The ‘*' shown for the intermediates indicates that the structure of P5abc or P5c in the intermediate may differ slightly from their corresponding fully unfolded or folded forms. Rate constants of the fast step in path #2 were derived from the *R*_1*ρ*_ data in the presence of 1 mM Mg^2+^ at 10 °C (see Fig. 2c). The overall *k*_obs_ for folding is obtained from a prior study^40^ in the presence of 0.5 mM Mg^2+^ at 10 °C.

Previous studies reported that mutations that stabilize tP5abc^Nat^ do not significantly affect the overall rate of folding^40^. It was proposed that P5c switches following a rate-limiting step, and is coupled to the formation of tertiary interactions^40^ (Fig. 7 path #1). Given its high modularity, our results would predict that P5c switching would remain fast even after other parts of the molecule (P5a and P5b) have partially folded.

In prior studies, early P5c switching was considered less likely since this could incur a very large activation barrier that could preclude P5abc from folding if this were the only available path^26^,^40^,^45^. Our results show that early P5c switching is rapid, with both forward and backward rate constants that are orders of magnitude larger than the overall rate of folding. Thus, folding could proceed via an alternative pathway (path #2) in which P5c switches early and yet still give rise to the previously observed single-exponential kinetics^40^ (Fig. 7). It is possible that different conditions (for example, Mg^2+^ concentration) can change the flux through these different folding pathways. Additional kinetic experiments will ultimately be needed to assess the extent of folding via these and other folding pathways.

## Discussion

NMR RD studies have provided great insights into fast-forming protein folding intermediates culminating in the high-resolution structure determination of these short-lived, low-populated species^10^,^11^,^12^. Our study shows that ^15^N NMR RD methods, when combined with chemical probing and mutagenesis, can be applied to study fast-forming intermediates during RNA tertiary folding and provide rare information regarding their structure. Recent advances that permit use of NMR RD to directly measure 3D structural information^46^ when combined with mutagenesis to trap the ES^13^,^14^,^15^,^16^, should enable the high-resolution 3D structure determination of RNA intermediates.

RNA structure is highly modular, and many structural motifs can form independent of tertiary context^47^. The P5c switch characterized in this work extends this modularity to dynamics—the backward and forward rate constants for P5c switching are highly independent of tertiary context and presence/absence of Mg^2+^. Such rapid modular secondary structural switches may be quite common during RNA tertiary folding and ribonucleoprotein assembly where they may help smoothen the folding landscape by helping break down the tertiary folding into a series of kinetically labile microscopic steps. This dynamic modularity may also be utilized in biological switches and should be considered in the design of RNA-based devices^48^.

The partially folded RNA intermediate identified in our study contains a specific hairpin (P5c) that independently adopts a native secondary structure (Fig. 2a). Interestingly, our data suggest that the intermediate is further stabilized by non-native interactions, the WC A173–U167 base pair and G176·G164 mispair that do not exist in either the GS or ES (Fig. 2a). Non-native interactions have previously been shown to stabilize protein folding intermediates^10^,^49^, and non-native base pairs are also quite common during RNA tertiary folding. RNA non-native structural elements can be quite extensive, involving entire elements of secondary structure, and thus melting of these non-native base pairs can be energetically costly and lead to the common observation of kinetically trapped intermediates that slow tertiary folding^50^,^51^,^52^,^53^,^54^,^55^,^56^,^57^,^58^. In contrast, the short-lived low-abundance ES intermediates characterized in this work, feature fewer non-native base pairs that can more easily be disrupted during tertiary folding. By helping break down the tertiary folding into multiple kinetically labile microscopic steps, they can help to ensure smooth and rapid tertiary folding.

## Methods

### NMR sample preparation

Uniformly ^13^C-/^15^N-labelled RNA samples were prepared by *in vitro* transcription using ^13^C-/^15^N-labelled nucleotide triphosphates (Cambridge Isotope Laboratories), T7 RNA polymerase (Fisher Scientific), and chemically synthesized DNA templates containing T7 promoter at 5′-end (Integrated DNA Technologies). The tP5abc^G176A^ sample was purified by ion-exchange chromatography using HPLC (Alliance, Waters) and exchanged into NMR buffer (10 mM sodium phosphate, 0.01 mM EDTA, pH 6.4) using 3-kDa-cutoff centrifugal concentrators (Millipore Corp.). The sample was refolded by heating at 95 °C for 10 min in 2 ml NMR buffer and rapidly cooled on ice and concentrated to ∼0.25 ml. All the other RNA samples were purified using 20% (w/v) denaturing polyacrylamide gel electrophoresis (PAGE) in 8 M urea and 1 × TBE buffer, followed by electro-elution and ethanol precipitation. Samples purified by PAGE were dissolved in water and were refolded in the same way, followed by buffer exchange into NMR buffer (10 mM sodium phosphate, 0.01 mM EDTA, pH 6.4).

### NMR spectroscopy

All NMR data were collected at 10 °C on Bruker Avance 600 MHz spectrometer or on Bruker Avance 700 MHz spectrometer both equipped with 5 mm triple-resonance cryogenic probes.

*Resonance assignments*. Imino resonances were assigned using 2D ^15^N-edited ^1^H-^1^H NOESY homonuclear correlation experiments with 120 ms mixing time and 2D imino ^1^H-^15^N HSQC experiments. All data were processed and analysed using the software NMRPipe^59^ and Sparky^60^, respectively.

*^15^N R_1ρ_ RD*. All 1D ^15^N *R*_1*ρ*_ RD experiments^44^ targeting imino nitrogen resonances were carried out at 14.1 T (except for data shown in Fig. 2c which were collected at 16.4 T) at 10 °C. On- and off-resonance *R*_1*ρ*_ RD profiles were recorded using spin-lock powers (*ω*_SL_/2*π*) ranging from 100 to 2,000 Hz and 100 to 300 Hz, respectively, with the absolute offset frequencies (*Ω*/2*π*) ranging from 0 to 3.5 × the applied spin-lock power (Supplementary Table 1). Magnetization of the spins of interest was allowed to relax under an applied spin-lock field for the following durations: (0–60 ms) for N1/N3 in tP5abc and mutants, and (0–150 ms) for N1/N3 in iP5c. Data were processed using NMRPipe^59^ to generate a series of peak intensities.

### Analysis of R_1ρ_ data

*R*_1*ρ*_ values were calculated by fitting the decay of peak intensity versus relaxation delay to a monoexponential. Errors in *R*_1*ρ*_ were estimated using a Monte Carlo-based approach with 50 iterations. Measured on- and off-resonance *R*_1*ρ*_ data were globally fit to the Laguerre equation (equation (1)) of two-site chemical exchange^61^ weighted to the experimental error in the *R*_1*ρ*_ data.


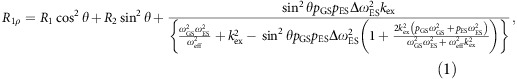


where, 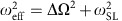, 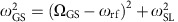, 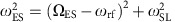, 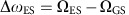, 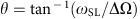, 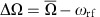; *R*_1_ and *R*_2_ are the longitudinal and transverse relaxation rates, respectively, Ω_GS_ and Ω_ES_ are the resonance offsets from the spin-lock carrier for the respective states, *ω*_rf_ is the reference frequency, *ω*_SL_ is the strength of the spin-lock carrier, 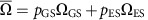 and *k*_ex_ is the exchange rate.

^15^N RD data were fitted globally by sharing *k*_ex_ and *p*_B_. The errors in best-fit parameters were estimated by a Monte Carlo approach with 50 iterations. The validity of the Laguerre equation under this particular exchange regime was established using Bloch–McConnell simulations^62^ (Supplementary Fig. 3c).

### SHAPE footprinting

RNA constructs were generated and purified from DNA templates containing a 5′ T7 promoter by fragment-assembly PCR of oligonucleotides (Integrated DNA Technologies)^63^. RNA was transcribed with T7 RNA polymerase and purified by affinity column (QIAGEN). P5abc RNA (0.2 μM) was folded for 15 min at 37 °C in 0 or 10 mM MgCl_2_ and NMR buffer containing 10 mM sodium phosphate and 0.01 mM EDTA (pH 6.4). A pre-denaturation step of the RNA for 2 min at 95 °C did not change the obtained results. RNA was equilibrated to 10 °C for at least 30 min; longer incubations up to 120 min did not change the results for WT P5abc. RNAs were incubated with 4.2 mg ml^−1^ 1M7 (1-methyl-7-nitroisatoic anhydride) in anhydrous DMSO (final DMSO 5%) for 60 min followed by purification and reverse transcription^63^. Nucleotides that do not participate in base pairing are selectively modified at the 2′-OH moiety which gives rise to reverse-transcription stops at the modified residues. cDNA fragments were resolved by capillary electrophoresis on a AB 3730 sequencer (Applied Biosystems).

### Analysis of SHAPE footprinting data

Alignment and quantitative analysis of electrophoretic traces was carried out in HiTRACE^64^. To rigorously compare reactivities between different P5abc mutants, each RNA construct included the P5abc sequence flanked by 5′- and 3′-hairpins separated by single-stranded buffer regions. The reactivity data were normalized to the single-stranded nucleotides in the 3′-end of the P5abc sequence^65^. The average reactivity of loop nucleotides in the 5′-hairpin across all experiments is 0.96±0.05 relative to the 3′-hairpin average, supporting the use of the hairpins as internal controls for SHAPE reactivity. The error in reactivity at each position was obtained from the s.e.m. of at least two measurements.

## Additional information

**How to cite this article:** Xue, Y. *et al.* Visualizing the formation of an RNA folding intermediate through a fast highly modular secondary structure switch. *Nat. Commun.* 7:11768 doi: 10.1038/ncomms11768 (2016).

## Supplementary Material

Supplementary Information Supplementary Figures 1-6 and Supplementary Tables 1-4.

## References

[b1] Herschlag D. RNA chaperones and the RNA folding problem. J. Biol. Chem. 270, 20871–20874 (1995). 7545662 10.1074/jbc.270.36.20871

[b2] Shajani Z., Sykes M. T. & Williamson J. R. Assembly of bacterial ribosomes. Annu. Rev. Biochem. 80, 501–526 (2011). 21529161 10.1146/annurev-biochem-062608-160432

[b3] Woodson S. A. Compact intermediates in RNA folding. Annu. Rev. Biophys. 39, 61–77 (2010). 20192764 10.1146/annurev.biophys.093008.131334 PMC6341483

[b4] Aleman E. A., Lamichhane R. & Rueda D. Exploring RNA folding one molecule at a time. Curr. Opin. Chem. Biol. 12, 647–654 (2008). 18845269 10.1016/j.cbpa.2008.09.010

[b5] Sclavi B., Sullivan M., Chance M. R., Brenowitz M. & Woodson S. A. RNA folding at millisecond intervals by synchrotron hydroxyl radical footprinting. Science 279, 1940–1943 (1998). 9506944 10.1126/science.279.5358.1940

[b6] Mulder A. M. *et al.* Visualizing ribosome biogenesis: parallel assembly pathways for the 30S subunit. Science 330, 673–677 (2010). 21030658 10.1126/science.1193220 PMC2990404

[b7] Talkington M. W., Siuzdak G. & Williamson J. R. An assembly landscape for the 30S ribosomal subunit. Nature 438, 628–632 (2005). 16319883 10.1038/nature04261 PMC1444899

[b8] Korzhnev D. M. & Kay L. E. Probing invisible, low-populated States of protein molecules by relaxation dispersion NMR spectroscopy: an application to protein folding. Acc. Chem. Res. 41, 442–451 (2008). 18275162 10.1021/ar700189y

[b9] Palmer A. G. 3rd Chemical exchange in biomacromolecules: Past, present, and future. J. Magn. Reson. 241, 3–17 (2014). 24656076 10.1016/j.jmr.2014.01.008 PMC4049312

[b10] Korzhnev D. M., Religa T. L., Banachewicz W., Fersht A. R. & Kay L. E. A transient and low-populated protein-folding intermediate at atomic resolution. Science 329, 1312–1316 (2010). 20829478 10.1126/science.1191723

[b11] Meinhold D. W. & Wright P. E. Measurement of protein unfolding/refolding kinetics and structural characterization of hidden intermediates by NMR relaxation dispersion. Proc. Natl Acad. Sci. USA 108, 9078–9083 (2011). 21562212 10.1073/pnas.1105682108 PMC3107309

[b12] Cho J. H., O'Connell N., Raleigh D. P. & Palmer A. G. 3rd Phi-value analysis for ultrafast folding proteins by NMR relaxation dispersion. J. Am. Chem. Soc. 132, 450–451 (2010). 20028088 10.1021/ja909052h PMC2860800

[b13] Blad H., Reiter N. J., Abildgaard F., Markley J. L. & Butcher S. E. Dynamics and metal ion binding in the U6 RNA intramolecular stem-loop as analyzed by NMR. J. Mol. Biol. 353, 540–555 (2005). 16181635 10.1016/j.jmb.2005.08.030

[b14] Dethoff E. A., Petzold K., Chugh J., Casiano-Negroni A. & Al-Hashimi H. M. Visualizing transient low-populated structures of RNA. Nature 491, 724–728 (2012). 23041928 10.1038/nature11498 PMC3590852

[b15] Zhao B. & Zhang Q. Characterizing excited conformational states of RNA by NMR spectroscopy. Curr. Opin. Struct. Biol. 30, 134–146 (2015). 25765780 10.1016/j.sbi.2015.02.011 PMC4755334

[b16] Xue Y. *et al.* Characterizing RNA excited states using NMR relaxation dispersion. Methods Enzymol. 558, 39–73 (2015). 26068737 10.1016/bs.mie.2015.02.002 PMC4756924

[b17] Hoogstraten C. G., Wank J. R. & Pardi A. Active site dynamics in the lead-dependent ribozyme. Biochemistry 39, 9951–9958 (2000). 10933815 10.1021/bi0007627

[b18] Johnson J. E. Jr. & Hoogstraten C. G. Extensive backbone dynamics in the GCAA RNA tetraloop analyzed using 13C NMR spin relaxation and specific isotope labeling. J. Am. Chem. Soc. 130, 16757–16769 (2008). 19049467 10.1021/ja805759z PMC2729180

[b19] Lee J., Dethoff E. A. & Al-Hashimi H. M. Invisible RNA state dynamically couples distant motifs. Proc. Natl Acad. Sci. USA 111, 9485–9490 (2014). 24979799 10.1073/pnas.1407969111 PMC4084437

[b20] Micura R. & Hobartner C. On secondary structure rearrangements and equilibria of small RNAs. Chembiochem. 4, 984–990 (2003). 14523915 10.1002/cbic.200300664

[b21] Furtig B. *et al.* Conformational dynamics of bistable RNAs studied by time-resolved NMR spectroscopy. J. Am. Chem. Soc. 129, 16222–16229 (2007). 18047344 10.1021/ja076739r

[b22] Rajkowitsch L. *et al.* RNA chaperones, RNA annealers and RNA helicases. RNA. Biol. 4, 118–130 (2007). 18347437 10.4161/rna.4.3.5445

[b23] Czernek J., Fiala R. & Sklenar V. Hydrogen bonding effects on the 15N and 1H shielding tensors in nucleic acid base pairs. J. Magn. Reson. 145, 142–146 (2000). 10873505 10.1006/jmre.2000.2091

[b24] Cate J. H. *et al.* Crystal structure of a group I ribozyme domain: principles of RNA packing. Science 273, 1678–1685 (1996). 8781224 10.1126/science.273.5282.1678

[b25] Murphy F. L., Wang Y. H., Griffith J. D. & Cech T. R. Coaxially stacked RNA helices in the catalytic center of the Tetrahymena ribozyme. Science 265, 1709–1712 (1994). 8085157 10.1126/science.8085157

[b26] Wu M. & Tinoco I. Jr. RNA folding causes secondary structure rearrangement. Proc. Natl Acad. Sci. USA 95, 11555–11560 (1998). 9751704 10.1073/pnas.95.20.11555 PMC21679

[b27] Golden B. L., Gooding A. R., Podell E. R. & Cech T. R. A preorganized active site in the crystal structure of the Tetrahymena ribozyme. Science 282, 259–264 (1998). 9841391 10.1126/science.282.5387.259

[b28] Russell R. & Herschlag D. New pathways in folding of the Tetrahymena group I RNA enzyme. J. Mol. Biol. 291, 1155–1167 (1999). 10518951 10.1006/jmbi.1999.3026

[b29] Treiber D. K., Rook M. S., Zarrinkar P. P. & Williamson J. R. Kinetic intermediates trapped by native interactions in RNA folding. Science 279, 1943–1946 (1998). 9506945 10.1126/science.279.5358.1943

[b30] Zarrinkar P. P. & Williamson J. R. Kinetic intermediates in RNA folding. Science 265, 918–924 (1994). 8052848 10.1126/science.8052848

[b31] Mitchell D. 3rd & Russell R. Folding pathways of the Tetrahymena ribozyme. J. Mol. Biol. 426, 2300–2312 (2014). 24747051 10.1016/j.jmb.2014.04.011 PMC4177526

[b32] Wan Y., Suh H., Russell R. & Herschlag D. Multiple unfolding events during native folding of the Tetrahymena group I ribozyme. J. Mol. Biol. 400, 1067–1077 (2010). 20541557 10.1016/j.jmb.2010.06.010 PMC2905490

[b33] Laederach A., Shcherbakova I., Jonikas M. A., Altman R. B. & Brenowitz M. Distinct contribution of electrostatics, initial conformational ensemble, and macromolecular stability in RNA folding. Proc. Natl Acad. Sci. USA 104, 7045–7050 (2007). 17438287 10.1073/pnas.0608765104 PMC1855354

[b34] Kwok L. W. *et al.* Concordant exploration of the kinetics of RNA folding from global and local perspectives. J. Mol. Biol. 355, 282–293 (2006). 16303138 10.1016/j.jmb.2005.10.070

[b35] Das R. *et al.* The fastest global events in RNA folding: electrostatic relaxation and tertiary collapse of the Tetrahymena ribozyme. J. Mol. Biol. 332, 311–319 (2003). 12948483 10.1016/s0022-2836(03)00854-4

[b36] Russell R. *et al.* Exploring the folding landscape of a structured RNA. Proc. Natl Acad. Sci. USA. 99, 155–160 (2002). 11756689 10.1073/pnas.221593598 PMC117531

[b37] Russell R. & Herschlag D. Probing the folding landscape of the Tetrahymena ribozyme: commitment to form the native conformation is late in the folding pathway. J. Mol. Biol. 308, 839–851 (2001). 11352576 10.1006/jmbi.2001.4751

[b38] Zheng M., Wu M. & Tinoco I. Jr. Formation of a GNRA tetraloop in P5abc can disrupt an interdomain interaction in the Tetrahymena group I ribozyme. Proc. Natl Acad. Sci. USA 98, 3695–3700 (2001). 11274387 10.1073/pnas.051608598 PMC31114

[b39] Silverman S. K., Zheng M., Wu M., Tinoco I. Jr. & Cech T. R. Quantifying the energetic interplay of RNA tertiary and secondary structure interactions. RNA 5, 1665–1674 (1999). 10606276 10.1017/s1355838299991823 PMC1369887

[b40] Koculi E., Cho S. S., Desai R., Thirumalai D. & Woodson S. A. Folding path of P5abc RNA involves direct coupling of secondary and tertiary structures. Nucleic Acids Res. 40, 8011–8020 (2012). 22641849 10.1093/nar/gks468 PMC3439887

[b41] Low J. T. & Weeks K. M. SHAPE-directed RNA secondary structure prediction. Methods 52, 150–158 (2010). 20554050 10.1016/j.ymeth.2010.06.007 PMC2941709

[b42] Korzhnev D. M., Orekhov V. Y. & Kay L. E. Off-resonance R1rho NMR studies of exchange dynamics in proteins with low spin-lock fields: An application to a fyn SH3 domain. J. Am. Chem. Soc. 127, 713–721 (2005). 15643897 10.1021/ja0446855

[b43] Hansen A. L., Nikolova E. N., Casiano-Negroni A. & Al-Hashimi H. M. Extending the range of microsecond-to-millisecond chemical exchange detected in labeled and unlabeled nucleic acids by selective carbon R1rho NMR spectroscopy. J. Am. Chem. Soc. 131, 3818–3819 (2009). 19243182 10.1021/ja8091399

[b44] Nikolova E. N., Gottardo F. L. & Al-Hashimi H. M. Probing transient Hoogsteen hydrogen bonds in canonical duplex DNA using NMR relaxation dispersion and single-atom substitution. J. Am. Chem. Soc. 134, 3667–3670 (2012). 22309937 10.1021/ja2117816 PMC3791138

[b45] Thirumalai D. Native secondary structure formation in RNA may be a slave to tertiary folding. Proc. Natl Acad. Sci. USA 95, 11506–11508 (1998). 9751694 10.1073/pnas.95.20.11506 PMC33899

[b46] Zhao B. & Zhang Q. Measuring residual dipolar couplings in excited conformational states of nucleic acids by CEST NMR spectroscopy. J. Am. Chem. Soc. 137, 13480–13483 (2015). 26462068 10.1021/jacs.5b09014 PMC4664061

[b47] Moore P. B. Structural motifs in RNA. Annu. Rev. Biochem. 68, 287–300 (1999). 10872451 10.1146/annurev.biochem.68.1.287

[b48] Chang A. L., Wolf J. J. & Smolke C. D. Synthetic RNA switches as a tool for temporal and spatial control over gene expression. Curr. Opin. Biotechnol. 23, 679–688 (2012). 22305712 10.1016/j.copbio.2012.01.005 PMC3354030

[b49] Klein-Seetharaman J. *et al.* Long-range interactions within a nonnative protein. Science 295, 1719–1722 (2002). 11872841 10.1126/science.1067680

[b50] Russell R. RNA misfolding and the action of chaperones. Front. Biosci. 13, 1–20 (2008). 17981525 10.2741/2557 PMC2610265

[b51] Chadalavada D. M., Knudsen S. M., Nakano S. & Bevilacqua P. C. A role for upstream RNA structure in facilitating the catalytic fold of the genomic hepatitis delta virus ribozyme. J. Mol. Biol. 301, 349–367 (2000). 10926514 10.1006/jmbi.2000.3953

[b52] Madore E., Florentz C., Giege R. & Lapointe J. Magnesium-dependent alternative foldings of active and inactive *Escherichia coli* tRNA(Glu) revealed by chemical probing. Nucleic Acids Res. 27, 3583–3588 (1999). 10446250 10.1093/nar/27.17.3583 PMC148604

[b53] Treiber D. K. & Williamson J. R. Exposing the kinetic traps in RNA folding. Curr. Opin. Struct. Biol. 9, 339–345 (1999). 10361090 10.1016/S0959-440X(99)80045-1

[b54] Pan T. & Sosnick T. R. Intermediates and kinetic traps in the folding of a large ribozyme revealed by circular dichroism and UV absorbance spectroscopies and catalytic activity. Nat. Struct. Biol. 4, 931–938 (1997). 9360610 10.1038/nsb1197-931

[b55] Woodson S. A. & Cech T. R. Alternative secondary structures in the 5' exon affect both forward and reverse self-splicing of the Tetrahymena intervening sequence RNA. Biochemistry 30, 2042–2050 (1991). 1998665 10.1021/bi00222a006

[b56] Cole P. E., Yang S. K. & Crothers D. M. Conformational changes of transfer ribonucleic acid. Equilibrium phase diagrams. Biochemistry 11, 4358–4368 (1972). 4562590 10.1021/bi00773a024

[b57] Ishida T. & Sueoka N. Rearrangement of the secondary structure of the secondary structure of tryptophan sRNA in *Escherichia coli*. Proc. Natl Acad. Sci. USA 58, 1080–1087 (1967). 4861304 10.1073/pnas.58.3.1080 PMC335750

[b58] Lindahl T., Adams A. & Fresco J. R. Isolation of ‘renaturable' transfer ribonucleic acids. J. Biol. Chem. 242, 3129–3134 (1967). 5338922

[b59] Delaglio F. *et al.* NMRPipe—a multidimensional spectral processing system based on Unix pipes. J. Biomol. NMR. 6, 277–293 (1995). 8520220 10.1007/BF00197809

[b60] Goddard T. D. & Kneller D. G. *SPARKY 3*, (University of California, San Franscisco).

[b61] Palmer A. G. 3rd & Massi F. Characterization of the dynamics of biomacromolecules using rotating-frame spin relaxation NMR spectroscopy. Chem. Rev. 106, 1700–1719 (2006). 16683750 10.1021/cr0404287

[b62] McConnell H. M. Reaction rates by nuclear magnetic resonance. J. Chem. Phys. 28, 430–431 (1958).

[b63] Cordero P., Kladwang W., VanLang C. C. & Das R. The mutate-and-map protocol for inferring base pairs in structured RNA. Methods. Mol. Biol. 1086, 53–77 (2014). 24136598 10.1007/978-1-62703-667-2_4 PMC4080707

[b64] Yoon S. *et al.* HiTRACE: high-throughput robust analysis for capillary electrophoresis. Bioinformatics 27, 1798–1805 (2011). 21561922 10.1093/bioinformatics/btr277

[b65] Kladwang W. *et al.* Standardization of RNA chemical mapping experiments. Biochemistry 53, 3063–3065 (2014). 24766159 10.1021/bi5003426 PMC4033625

[b66] Leontis N. B. & Westhof E. Geometric nomenclature and classification of RNA base pairs. RNA. 7, 499–512 (2001). 11345429 10.1017/s1355838201002515 PMC1370104

