# Supplementary material for: Visualizing the formation of an RNA folding intermediate through a fast highly modular secondary structure switch
Source: Nat Commun. 2016 Jun 13;7:ncomms11768. doi: 10.1038/ncomms11768 (PMC4909931; doi:10.1038/ncomms11768)
Supplement: Supplementary Information — Supplementary Figures 1-6 and Supplementary Tables 1-4. [file ncomms11768-s1.pdf]

**Supplementary Figure 1**

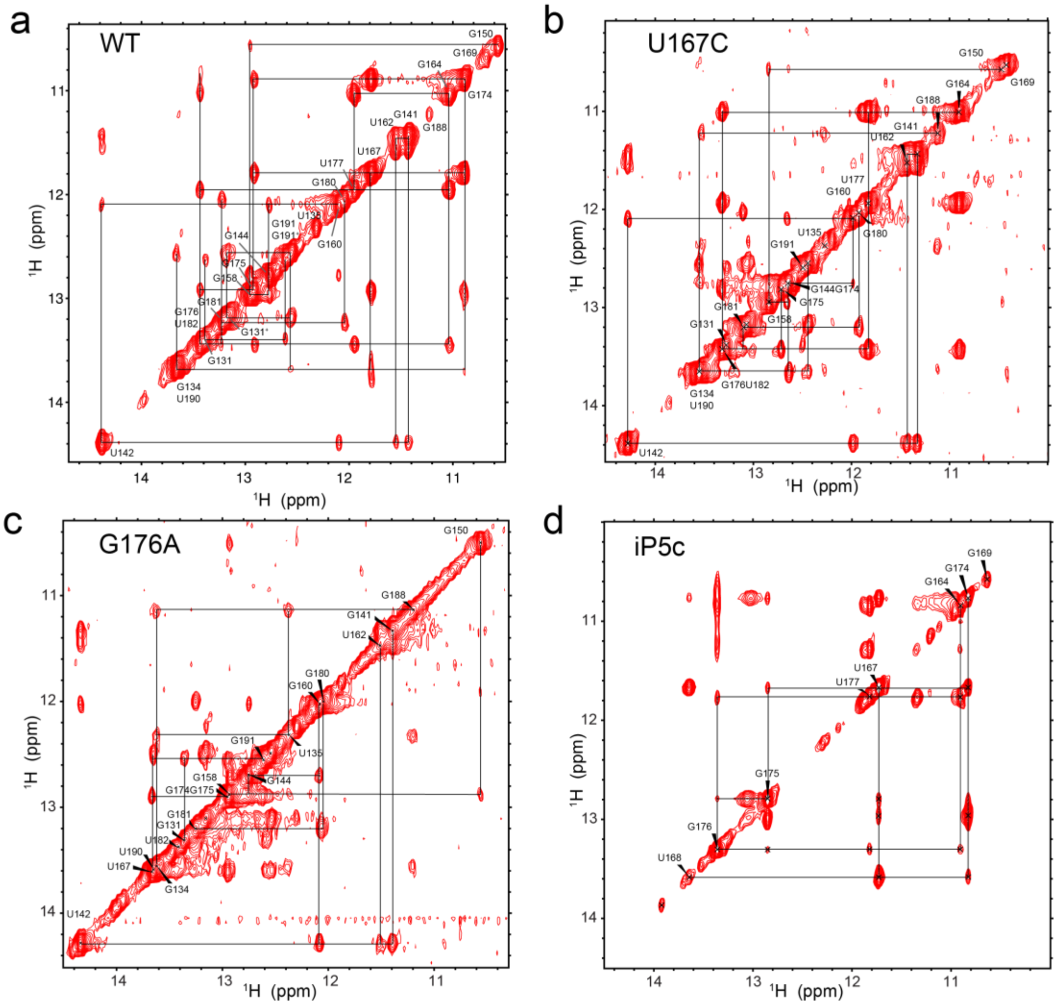


2D $^{15}$N-edited NOESY spectra showing NOE connectivities for (a) tP5abc, (b) tP5abc$^{U167C}$, (c) tP5abc$^{G176A}$, and (d) iP5c at 10 °C. In all cases, the buffer conditions were 10 mM sodium phosphate and 0.01 mM EDTA (pH 6.4). The RNA concentrations are 1 mM (tP5abc, tP5abc$^{U167C}$, and iP5c) and 2 mM (tP5abc$^{G176A}$). The spectra were recorded using mixing time 120 ms.

**Supplementary Figure 2**

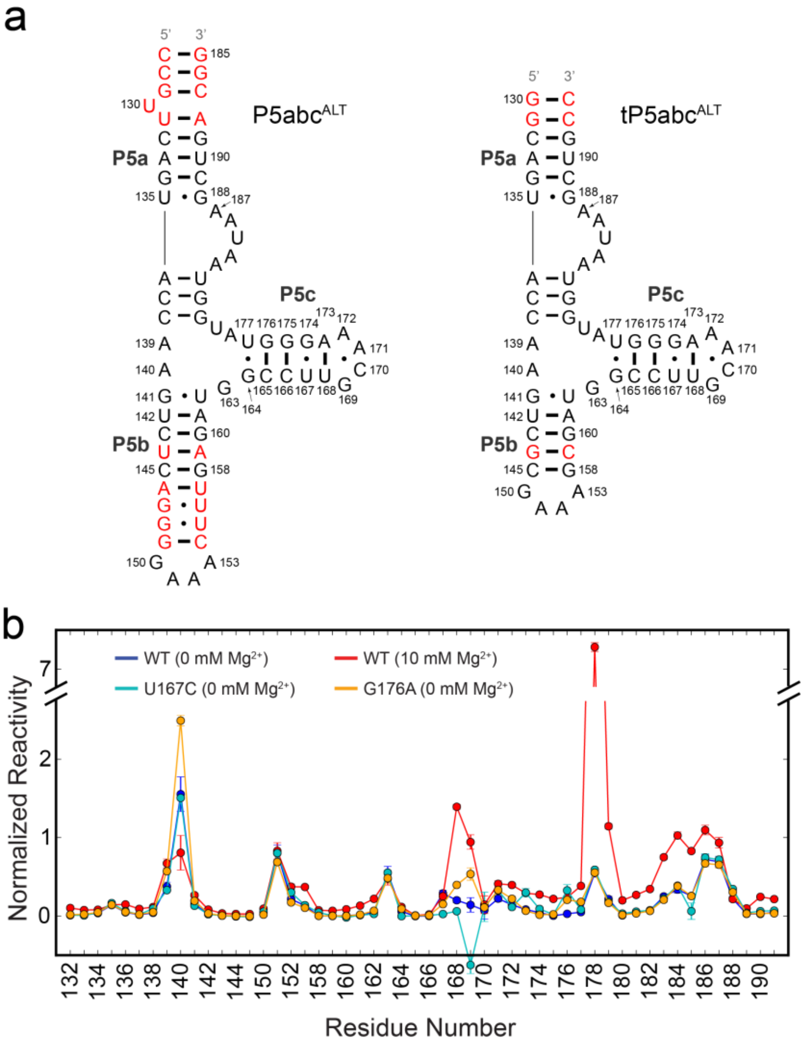


(a) Secondary structures of extended P5abc (left) used for SHAPE analysis and tP5abc (right) used for NMR study. The difference between P5abc and tP5abc are colored in red. (b) SHAPE normalized reactivity profiles of WT P5abc in the absence and the presence of 10 mM $Mg^{2+}$, and reactivity profiles of P5abc$^{U167C}$ and P5abc$^{G176A}$ in the absence of $Mg^{2+}$.

## Supplementary Figure 3

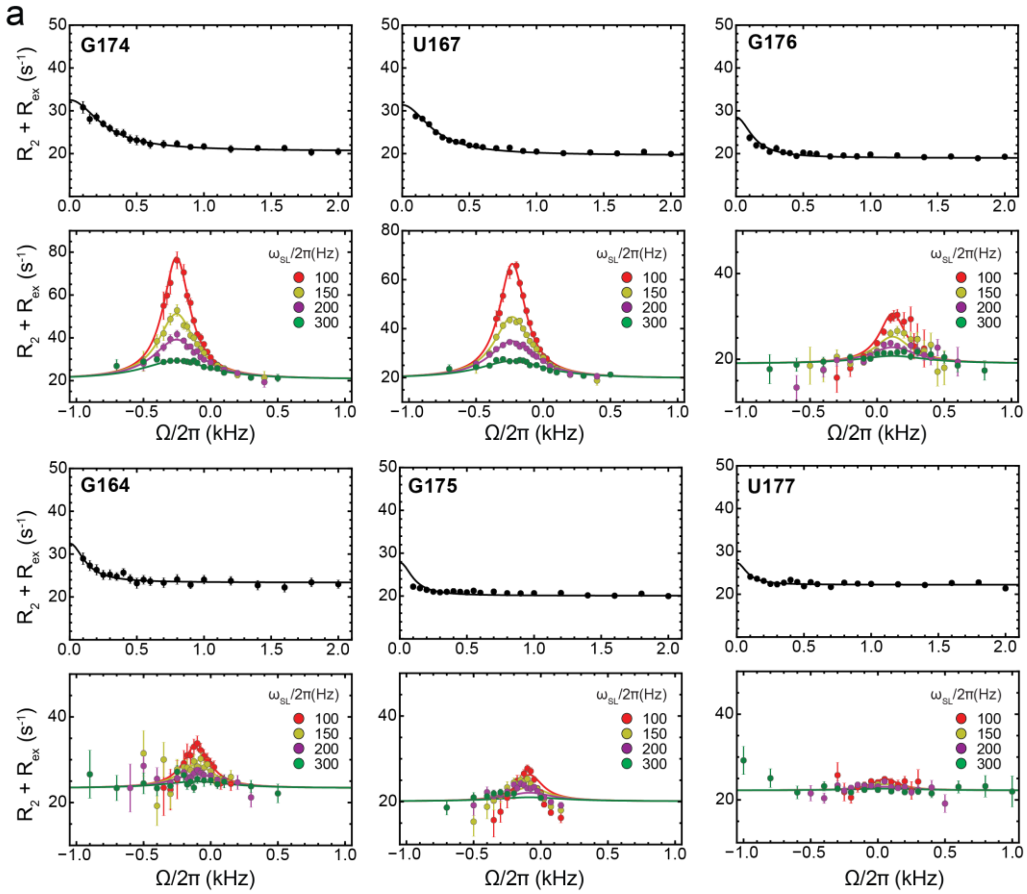

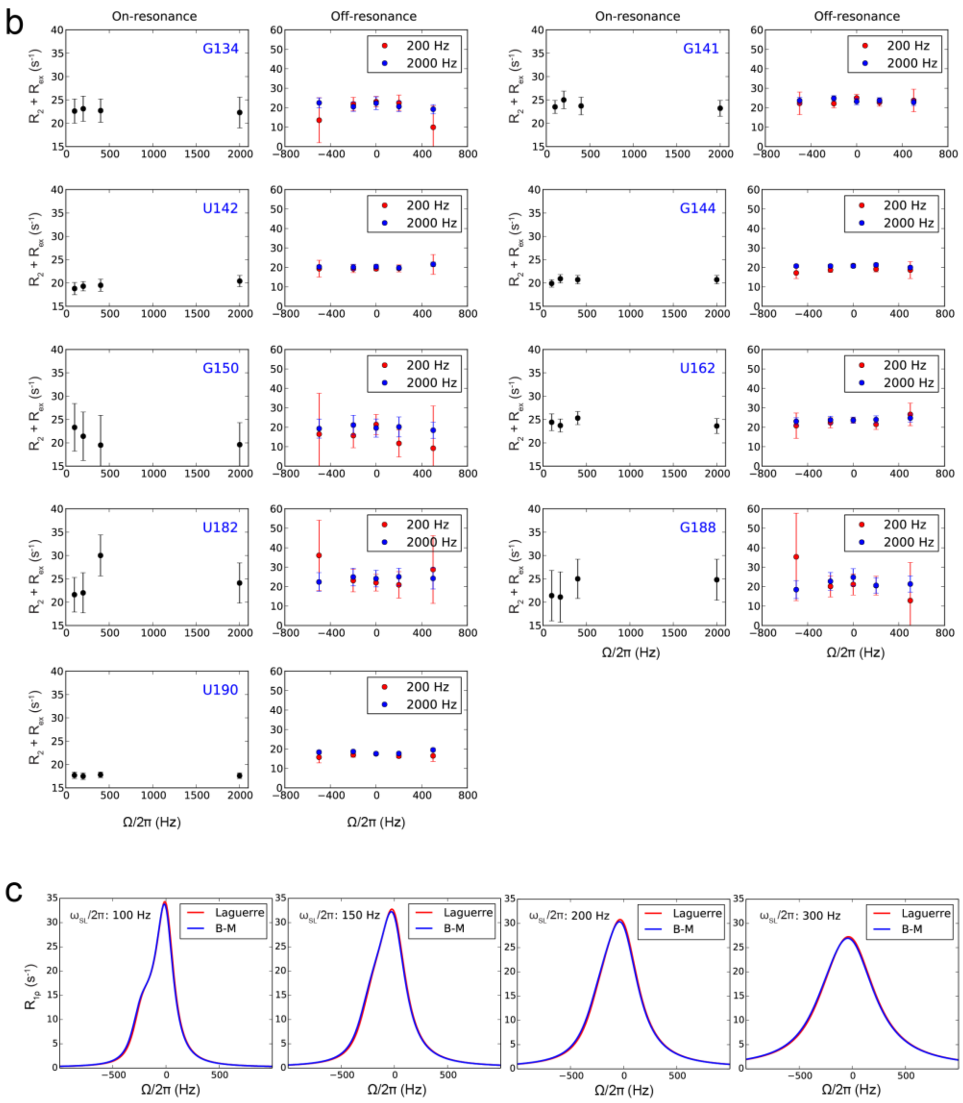


(a) On-resonance and off-resonance $^{15}$N RD profiles measured in well-resolved resonances belonging to P5c in tP5abc. (b) Additional on-resonance and off-resonance $^{15}$N RD profiles measured in tP5abc showing limited RD. Each $R_{1\rho}$ was measured using two time delays to reduce the acquisition time. $R_2$+$R_{ex}$ was calculated by setting $R_1$ = 0.5 s$^{-1}$ which represents the typical $R_1$ value for tP5abc. (c) Simulated $R_{1\rho}$ RD profiles as calculated using the Laguerre (red) and Bloch-McConnell (blue) equations. The

exchange parameters used for the simulation ($R_1$, $R_2$, $p_B$, $\Delta\omega$, $k_{ex}$) were obtained from fitting experimental data for G174 in tP5abc in the absence of $Mg^{2+}$.

**Supplementary Figure 4**

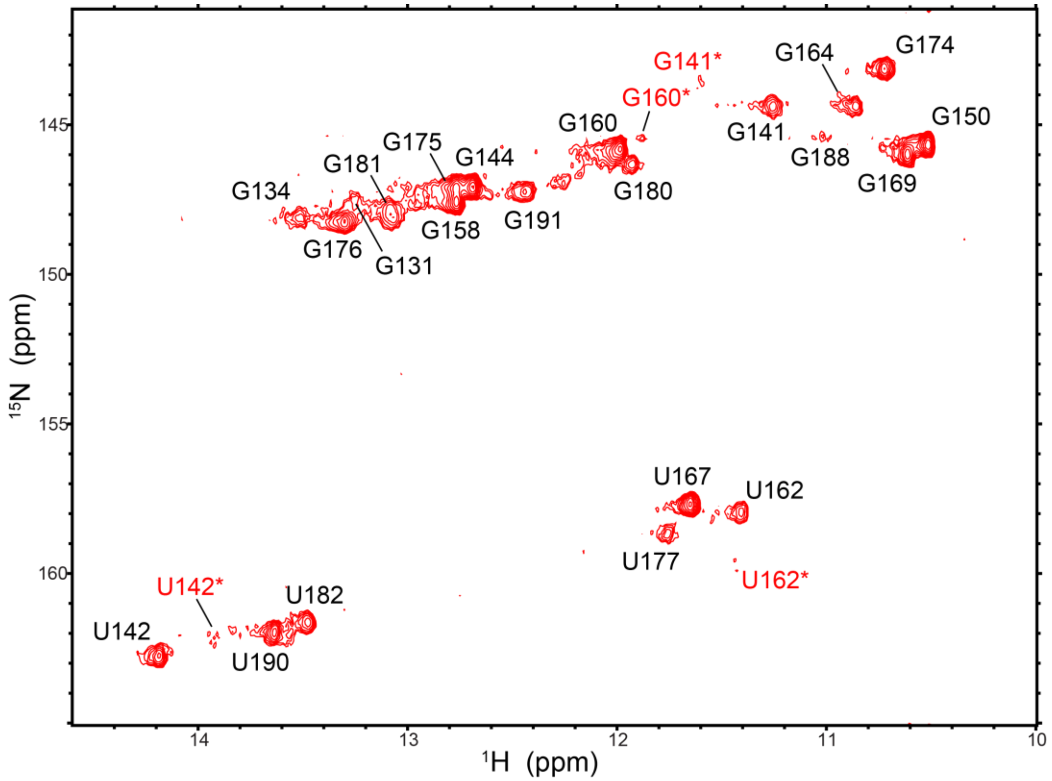


2D $^{1}H$-$^{15}N$ HSQC spectrum of tP5abc in 1 mM free $Mg^{2+}$ showing broadening of resonances. Resonances belonging to folded tP5abc$^{Nat}$ species are labeled with asterisk.

**Supplementary Figure 5**

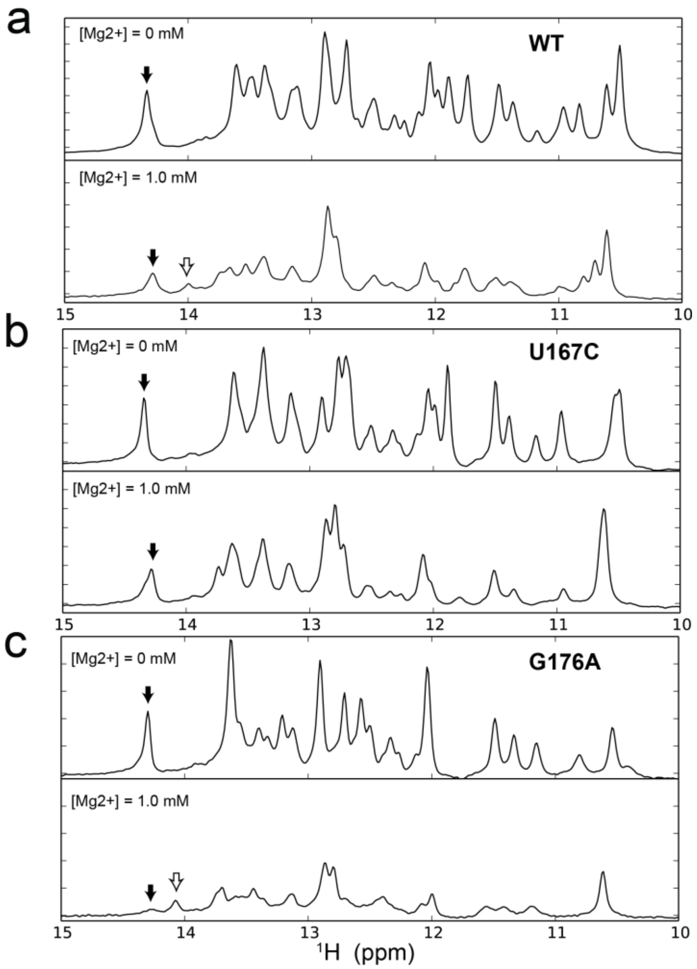


Monitoring $Mg^{2+}$ induced tertiary folding of (a) tP5abc, (b) $tP5abc^{U167C}$, and (c) $tP5abc^{G176A}$ using 1D imino SOFAST HMQC[31] at 10 °C. Highlighted resonances belong to U142 in $tP5abc^{Alt}$ (black arrow) and U142* in $tP5abc^{Nat}$ (white arrow). Samples were prepared by repeatedly exchanging RNA (0.1 mM) into NMR buffer (10 mM sodium phosphate and 0.01 mM EDTA at pH 6.4) containing the desired $Mg^{2+}$ concentration.

**Supplementary Figure 6**

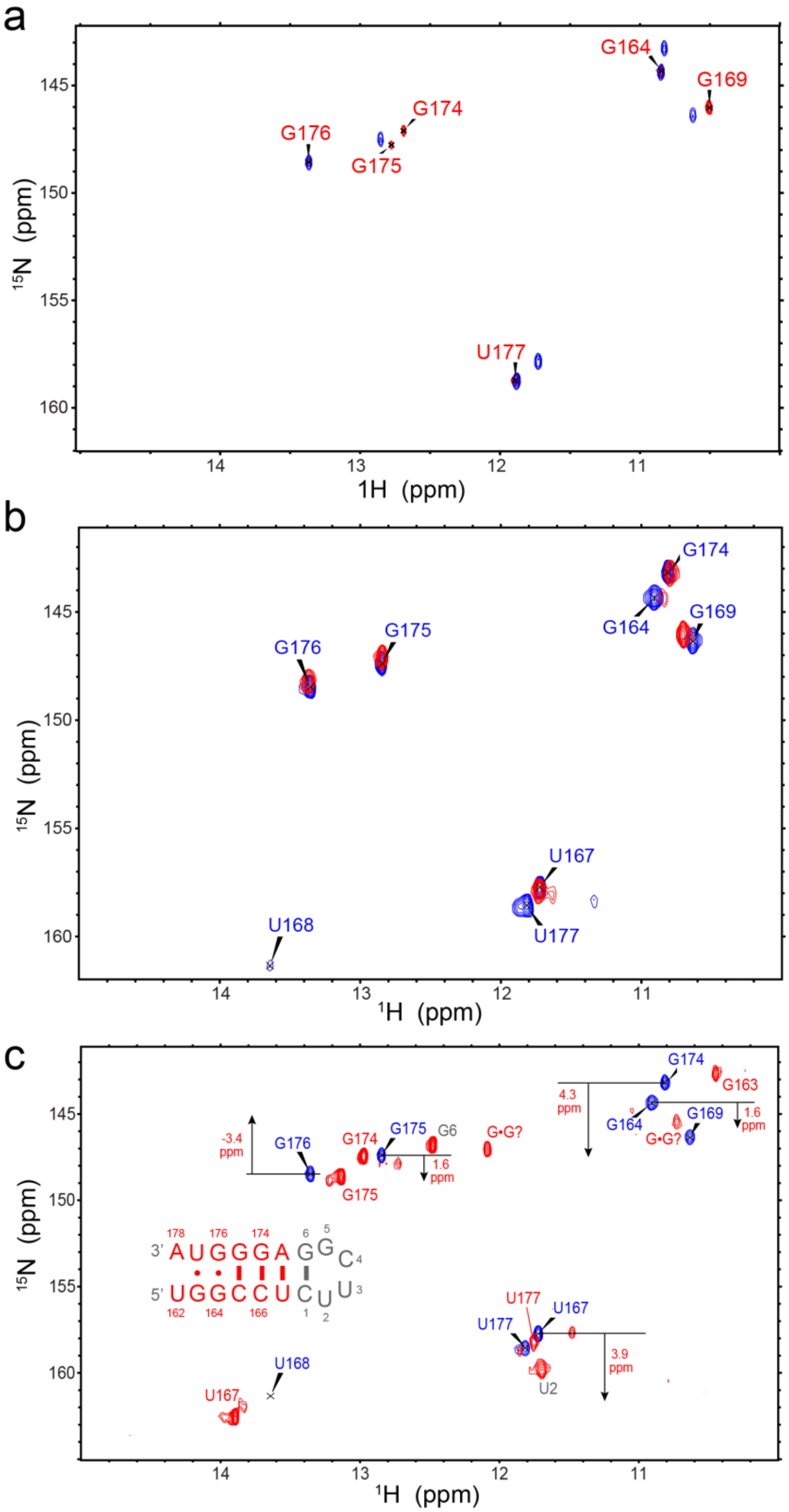

Additional spectra of iP5c and its variants. (a) Overlay of 2D imino SOFAST-HMQC spectrum of unlabeled iP5c$^{U167C}$ GS-mutant (red) with that of unlabeled iP5c (blue) in the absence of $Mg^{2+}$. (b) Imino SOFAST-HMQC spectra of 0.1 mM iP5c in the absence (blue) and the presence (red) of 30 mM $Mg^{2+}$. The magnesium ions were added into the sample directly. (c) Overlay of 2D imino SOFAST-HMQC spectrum of unlabeled iP5c$^{UUCG}$ ES-mutant (red) with that of iP5c (blue) in the absence of $Mg^{2+}$. The secondary structure of ES-mutant is shown in the inset. The residues showing pronounced RD in iP5c are highlighted by black arrows starting at $^{15}N$ chemical shift of GS and ending at $^{15}N$ chemical shift of ES, and are labeled with the associated $\Delta\omega$.

## Supplementary Table 1

Spin lock powers ($\omega_{SL}$) and offsets ($\Omega$) used in $R_{1\rho}$ relaxation dispersion experiments.

| **tP5abc** | **$\omega_{SL}/2\pi$ (Hz)** | **$\Omega/2\pi$ (Hz)** |
|---|---|---|
| **G174-N1 U167-N3** | 100 | -350, -325, -300, -250, -200, -175, -150, -125, ±100, -75, ±50, ±25 |
| | 150 | -500, ±400, -350, -300, -250, ±200, -175, -150, -125, ±100, -75, ±50 |
| | 200 | -500, ±400, -350, -300, -250, ±200, -175, -150, -125, ±100, -75, ±50 |
| | 300 | -700, ±500, -400, -350, ±300, -250, -200, -175, -150, -125, ±100, ±50 |
| **G176-N1** | 100 | ±50, ±100, 125, 150, ±200, 250, 275, ±300, 350 |
| | 150 | ±50, ±100, 150, ±200, 250, ±300, 350, ±400, 450, ±500 |
| | 200 | ±50, ±100, 150, ±200, 250, ±300, 350, ±400, 450, ±600 |
| | 300 | ±50, ±100, 150, ±200, 250, 300, ±400, 500, ±600, ±800 |
| **G164-N1** | 100 | -350, -300, -250, -200, -175, ±150, -125, -100, ±75, -50, ±25 |
| | 150 | -500, -400, -350, -300, -250, -200, -175, ±150, -125, -100, ±75, -50, ±25 |
| | 200 | -600, -500, -400, ±300, -250, ±200, -175, -150, -125, ±100, -75, ±50 |
| | 300 | -900, -700, ±500, -400, ±300, -250, -200, -175, -150, -125, ±100, ±50 |
| **U177-N3 G169-N1** | 100 | ±300, ±250, ±200, ±150, ±100, ±50 |
| | 200 | ±500, ±400, ±300, ±250, ±200, ±150, ±100, ±50 |
| | 300 | ±1000, ±800, ±600, ±400, ±300, ±200, ±100 |
| **G175-N1** | 100 | -350, -300, -250, -200, -175, ±150, -125, -100, ±75, -50, ±25 |
| | 150 | -500, -400, -350, -300, -250, -200, -175, ±150, -125, -100, ±75, -50, ±25 |
| | 200 | -500, -400, -350, -300, -250, -200, -175, ±150, -125, -100, ±75, -50, ±25 |
| | 300 | -700, -500, -400, -350, -300, -250, -200 |

| **iP5c** | **$\omega_{SL}/2\pi$ (Hz)** | **$\Omega/2\pi$ (Hz)** |
|---|---|---|
| **G174-N1** | 100 | -350, -325, -300, -250, -200, -175, -150, -125, ±100, -75, ±50, ±25 |
| | 150 | -500, ±400, -350, -300, -250, ±200, -175, -150, -125, ±100, -75, ±50 |
| | 200 | -500, ±400, -350, -300, -250, ±200, -175, -150, -125, ±100, -75, ±50 |
| | 300 | -700, ±500, -400, -350, ±300, -250, -200, -175, -150, -125, ±100, ±50 |
| **U167-N3** | 100 | -350, -325, -300, -275, -250, -200, -175, -150, -125, ±100, -75, ±50, ±25 |
| | 150 | -500, ±400, -350, -300, -250, ±200, -175, -150, -125, ±100, -75, ±50 |
| | 200 | -500, ±400, -350, -300, -250, ±200, -175, -150, -125, ±100, -75, ±50 |
| | 300 | -700, ±500, -400, -350, ±300, -250, -200, -175, -150, -125, ±100, ±50 |
| **G176-N1** | 100 | ±50, ±100, 125, 150, ±200, 250, 275, ±300, 350, 400 |
| | 150 | ±50, ±100, 150, ±200, 250, ±300, 350, ±400, 450, ±500 |
| | 200 | ±50, ±100, 150, ±200, 250, ±300, 350, ±400, 450, ±600 |
| | 300 | ±50, ±100, 150, ±200, 250, 300, ±400, 500, ±600, ±800 |

| | | |
|---|---|---|
| **G164-N1** **G175-N1** | 100 | -350, -300, -250, -200, -175, ±150, -125, -100, ±75, -50, ±25 |
| | 150 | -500, -400, -350, -300, -250, -200, -175, ±150, -125, -100, ±75, -50, ±25 |
| | 200 | -500, -400, -350, -300, -250, -200, -175, ±150, -125, -100, ±75, -50, ±25 |
| | 300 | -700, ±500, -400, -350, ±300, -250, -200, -175, -150, -125, ±100, ±50 |
| **U177-N3** **G169-N1** | 100 | ±300, ±250, ±200, ±150, ±100, ±50 |
| | 200 | ±500, ±400, ±300, ±250, ±200, ±150, ±100, ±50 |
| | 300 | ±1000, ±800, ±600, ±400, ±300, ±200, ±100 |

## Supplementary Table 2

Exchange parameters obtained from fitting $R_{1\rho}$ data measured in tP5abc and iP5c (in the absence of $Mg^{2+}$ at 10 °C) to two-state exchange model.

| **tP5abc** | $R_1$ ($s^{-1}$) | $R_2$ ($s^{-1}$) | $k_{ex}$ ($s^{-1}$) | $p_B$ | $\Delta\omega_{AB}$ (ppm) |
|---|---|---|---|---|---|
| **G174-N1** | 1.5±0.3 | 20.6±0.2 | | | 4.3±0.1 |
| **U167-N3** | 1.1±0.1 | 19.6±0.2 | | | 3.9±0.1 |
| **G176-N1** | 1.3±0.1 | 19.0±0.2 | | | -2.0±0.1 |
| **G164-N1** | 1.3±0.2 | 23.3±0.3 | 423 ± 26 | 2.9% ± 0.2% | 1.8±0.1 |
| **U177-N3** | 1.4±0.1 | 22.2±0.2 | | | N/A |
| **G169-N1** | 1.9±0.5 | 20.7±0.7 | | | N/A |
| **G175-N1** | 1.6±0.1 | 20.1±0.2 | | | 1.4±0.1 |

| **iP5c** | $R_1$ ($s^{-1}$) | $R_2$ ($s^{-1}$) | $k_{ex}$ ($s^{-1}$) | $p_B$ | $\Delta\omega_{AB}$ (ppm) |
|---|---|---|---|---|---|
| **G174-N1** | 2.9±0.1 | 9.3±0.1 | | | 4.3±0.1 |
| **U167-N3** | 2.7±0.1 | 9.0±0.1 | | | 3.9±0.1 |
| **G176-N1** | 2.6±0.1 | 8.7±0.1 | | | -3.4±0.1 |
| **G164-N1** | 2.5±0.1 | 10.4±0.1 | 323 ± 9 | 3.4%±0.1% | 1.6±0.1 |
| **U177-N3** | 2.5±0.1 | 9.9±0.1 | | | N/A |
| **G169-N1** | 2.2±0.1 | 8.4±0.1 | | | N/A |
| **G175-N1** | 2.4±0.1 | 9.2±0.1 | | | 1.6±0.1 |

## Supplementary Table 3

Exchange parameters obtained from fitting $R_{1\rho}$ data measured in tP5abc and iP5c (in the presence of 1 mM free $Mg^{2+}$ at 10 °C) to two-state exchange model.

| **tP5abc** | $R_1$ **(s$^{-1}$)** | $R_2$ **(s$^{-1}$)** | $k_{ex}$ **(s$^{-1}$)** | $p_B$ | $\Delta\omega_{AB}$ **(ppm)** |
|---|---|---|---|---|---|
| **G174-N1** | 0.8±0.3 | 22.1±0.4 | 329 ± 110 | 1.0% ± 0.3% | 3.9±0.4 |
| **U167-N3** | -0.7±0.2 | 22.2±0.3 | | | 4.1±0.3 |

| **iP5c** | $R_1$ **(s$^{-1}$)** | $R_2$ **(s$^{-1}$)** | $k_{ex}$ **(s$^{-1}$)** | $p_B$ | $\Delta\omega_{AB}$ **(ppm)** |
|---|---|---|---|---|---|
| **G174-N1** | 1.9±0.1 | 8.8±0.1 | 964 ± 67 | 0.52%±0.04% | 3.3±0.2 |
| **U167-N3** | 1.8±0.1 | 8.1±0.1 | | | 2.5±0.1 |

## Supplementary Table 4

Comparison of $\Delta\omega$ derived from $^{15}N$ RD experiments of tP5abc in the absence of $Mg^{2+}$ with $^{15}N$ chemical shift difference between $tP5abc^{G176A}$ and tP5abc (cf. Fig 4d).

| | $\Delta\omega_{AB}$(tP5abc) **(ppm)** | $\delta(tP5abc^{G176A}) - \delta(tP5abc)$ **(ppm)** |
|---|---|---|
| **G174-N1** | 4.3 ± 0.1 | 4.0 |
| **U167-N3** | 3.9 ± 0.1 | 3.6 |
| **G164-N1** | 1.8 ± 0.1 | 2.2 |
| **G175-N1** | 1.4 ± 0.1 | 1.2 [(a)] |
| **U135-N3** | 0.0 ± 0.1 | -0.1 |
| **G188-N1** | 0.0 ± 0.1 | 0.0 |

(a). Since G176A mutation introduces significant chemical shift perturbation to G175-N1, this number is obtained by calculating $^{15}N$ chemical shift difference between $iP5c^{UUCG}$ and iP5c (cf. Supplementary Fig. 6c).
